# Association between placental epigenetic age acceleration and early postnatal growth patterns

**DOI:** 10.1038/s41598-025-13951-y

**Published:** 2025-08-12

**Authors:** Priyadarshni Patel, Angela Shen, Cynthia Perez, Elizabeth M. Kennedy, Kartik Shankar, Kevin J. Pearson, Aline Andres, Todd M. Everson

**Affiliations:** 1 Gangarosa Department of Environmental Health, Rollins School of Public Health, Emory University, 1518 Clifton Road NE, 1518-002-2BB, Atlanta, GA 30322 USA; 2 Department of Gynecology and Obstetrics, School of Medicine, Emory University, Atlanta, GA USA; 3 Department of Human Genetics, School of Medicine, Emory University, Atlanta, GA USA; 4 Responsive Agricultural Food Systems Research Unit, USDA Agricultural Research Service, College Station, TX USA; 5 Department of Pharmacology and Nutritional Sciences, University of Kentucky, Lexington, KY USA; 6 Department of Pediatrics, University of Arkansas for Medical Sciences and Arkansas Children’s Nutrition Center, Little Rock, AR USA; 7 Department of Epidemiology, Rollins School of Public Health, Emory University, Atlanta, GA USA

**Keywords:** Epigenomics, Epigenetic age, Placenta, Growth, Metabolic health, Gestational age, Gestational age acceleration, Epigenetic age, CPC clock, Epigenetics, Environmental sciences

## Abstract

Placental gestational age acceleration (GAA) is the difference between the actual gestational age (GA) at birth and their estimated epigenetic gestational age (EGA), which is calculated from placental DNA methylation. Understanding the role of placental GAA in postnatal growth trajectories is crucial for early identification of infants at risk of altered growth patterns and associated long-term health outcomes. The objective of this study is to investigate the association between placental GAA and longitudinal growth trajectories specifically weight, height, fat mass, and lean mass gain in early childhood. This study uses placental DNA methylation at birth to calculate epigenetic GAA and longitudinal measures of weight, height, fat mass, and lean mass to generate growth trajectory characteristics. Higher placental GAA was significantly associated with slower weight gain (95% CI [− 0.03, − 0.001]) and fat mass (95% CI [− 0.08, − 0.02]) gain, as well as reduced average fat mass (95% CI [add this]) over the follow-up period. However, no significant associations were found between GAA and height or lean mass gain. Placental GAA can give early insights into altered postnatal growth trajectories, particularly for weight and fat mass where an increase in GAA is associated with decreased weight and fatmass gain over time while we observed no effect on height and lean mass. Understanding these associations offers insights into early developmental patterns and long-term health outcomes in children, highlighting the importance of perinatal factors in shaping growth trajectories in early childhood.

**Supplementary Information:**

The online version contains supplementary material available at 10.1038/s41598-025-13951-y.

## Introduction

The placenta performs many functions imperative for proper fetal growth and development as it facilitates nutrient and oxygen transport, hormone production, and supports processes including endocrine regulation, toxin and waste removal, as well as thermal regulation^1,2^. The biological activity and anatomical features of the placenta can be affected by maternal exposures and physiology, with consequences for offspring health outcomes^3^, including perturbed postnatal growth patterns^4^ and adiposity^5^. Postnatal growth patterns, specifically weight and height gain, are indicators for future health outcomes. Excessive growth, or rapid weight gain from infancy through mid-childhood, is consistently associated with later life obesity and increased cardiometabolic risk factors^6–12^.

Gestational factors that are informative about postnatal growth patterns can help us identify those at risk of childhood obesity and can help us understand how the *in utero* environment affects offspring growth and cardiometabolic health. Placental gestational age acceleration (GAA), the difference between a neonate’s actual gestational age (GA) at birth and their estimated epigenetic gestational age (EGA; a measure of biological maturity), may provide insights into how placental health or function relates to postnatal growth and adiposity. Four recently developed EGA clocks accurately estimate GA, with two using newborn cord blood DNAm profiles (Knight and Bohlin)^13,14^ and two using placental tissue DNAm profiles (Mayne and Lee)^15,16^. To date, the majority of studies have used cord blood to estimate and study gestational age acceleration^17,18^. Measuring GAA from placental tissue can help us better understand the interrelationships between the placenta, the prenatal environment and postnatal growth trajectories such as weight, height, fat mass and lean mass gains over time, which can potentially offer early insights into the timing and progression of various developmental milestones. With this study we aimed to test the associations between placental GAA and characteristics of weight, height, fat mass, and lean mass growth trajectory during the first two years of life.

## Methodology

### Study population

This study is a part of a Virtual Consortium for Translational/Transdisciplinary Environmental Research (ViCTER) funded by the National Institute of Environmental Health Sciences. ViCTER consortium’s project (R01 ES032176), using a longitudinal cohort (NCT03281850), where participants were recruited between 2010 and 2014 from central Arkansas. Eligibility criteria were maternal age over 21 years, either actively planning or already pregnant within the first 10 weeks of gestation, second parity singleton pregnancies, and BMI between 18.5 and 35 kg/m^2^. Exclusion criteria comprised preexisting medical conditions, sexually transmitted diseases (STDs), medication influencing fetal growth, substance use, and pregnancy complications. Gestational diabetes was an exclusion criterion in our study and therefore was not present in the analytic sample. Placentas with severe pathologies were excluded. The final cohort enrolled 300 pregnant women, with placental samples obtained from 152 participants at term within 30 min of delivery.

### Placental collection

All placental samples were collected within 30 min and processed within 2 h of delivery. After removing the umbilical cord and fetal membranes, samples of placental tissues were collected from the chorionic villous core following removal of the chorionic plate. The tissue underlying the chorionic plate consists of chorionic villous tissue and is of fetal origin. Samples were collected at 6 random sites (~ 1 sq. in) and washed thrice to remove maternal blood. To ensure comprehensive representation of the highly heterogenous placenta, ~1 g tissue from these 6 random sites was pooled, pulverized in liquid nitrogen and flash frozen. Samples were stored at -80 °C until DNA isolation.

### DNA methylation analysis and processing

Genomic DNA extraction was done with PureLink DNA isolation reagents (Thermo Fisher). DNA was quantified with Qubit dsDNA quantification assays (Thermo Fisher, Waltham, MA, USA), then aliquoted into standardized concentrations to allow for a total mass of 500 ng of DNA. Samples were randomly distributed across 96-well plates, rows, and chips to reduce the potential for batch effects. Bisulfite conversion was done with the EZ DNA Methylation Kit (Zymo Research, Irvine, CA) and CpG-specific DNAm was quantified using the Illumina MethylationEPIC Beadarray (Illumina, San Diego, CA) at the Emory Integrated Genomics Core. To reduce the technical variation and probe-type bias, functional normalization and beta mixture quantile normalization were used^19,20^. All samples passed quality control steps. There was no evidence of batch effect via principal component analysis.

### Gestational age acceleration

The epigenetic gestational age of 152 samples was estimated utilizing the R package planet^15^. This package comprises three distinct placental epigenetic clocks: the robust placenta clock (RPC), control placental clock (CPC), and refined RPC^15^. We focused on CPC due to its training on placental samples designated as controls, making it more representative of our study population compared to RPC, which was trained using methylation profiles from placentas with varied pregnancy conditions^15^. To generate GAA values, we regressed epigenetic GA on reported GA via linear regression, then extracted the residuals.

### Body measurements

Weight, height, fat mass, and lean mass gain were assessed at 11 postnatal time points (2 and 4 weeks, 2, 3, 4, 5, 6, 9, 12, 18 and 24 months) using standardized methodologies reported previously^21^. Child body composition was evaluated at 11 postnatal timepoints, using quantitative nuclear magnetic resonance (QMR, EchoMRI-AH small, Echo Medical System, Houston, TX), which was previously validated in this population^21^.

### Statistical analyses

All statistical analyses were conducted using R (version 4.2.2). We utilized the SuperImposition by Translation and Rotation (SITAR) model to characterize postnatal trajectories of weight, height, fat mass and lean mass from 2 weeks through 24 months of age. The SITAR model functions as a shape invariant framework that yields a mean curve alongside sets of three parameters per individual (size, tempo, and velocity), summarizing the individual growth trajectories^22^. Individual curves are aligned with the mean curve (across all participants) through vertical shifts (representing variations in mean *size*) and horizontal shifts (reflecting differences in age at peak growth velocity, or *tempo*). Additionally, adjustments (termed *intensity*) to the temporal scale capture individual variations in growth velocity. These three parameters are estimated as random effects for each study participant. The tempo parameter was not able to be generated from our data due to missing values, leaving intensity and size parameters for analysis. To date, SITAR has been commonly used to understand the growth trajectory of weight and height^23,24^, however, this study also used the same approach to demonstrate and understand the trajectories of fat mass and lean mass in children.

Size and intensity values for weight, height, fat mass and lean mass were regressed on GAA (independent variable) while adjusting for potential confounding variables that could affect the associations such as maternal age, maternal education, gestational age, gestational weight gain, maternal race, and placental cell type proportions. A statistical significance of 0.05 level was used for all the results.

## Results

The study sample is characterized in Table 1. Our sample included 42% males and 58% females with mean gestational age of 39.32 weeks. A majority of the mothers (70%) had a college degree and were White (86%). In our study sample, placental epigenetic age was highly correlated for all clocks, and was strongest for CPC (*r* = 0.641) and RPC (*r* = 0.641); CPC-derived GAA was utilized for all downstream analyses since the training data for CPC was most similar to our Glowing sample.

**Table 1:** General characteristics of the study population.

Parameter	
N	152
Child sex, N(%)	
Male	64 (42%)
Female	88 (58%)
Birth weight (kg)	3.53 ± 0.43
Birth length (cm)	51.30 ± 2.29
Gestational age (weeks)	39.32 ± 0.84
Maternal education, N (%)	
College degree	106 (70)
No college degree	46 (30)
Maternal race, N (%)	
Caucasian	131(86)
Non-Caucasian	21(14)
Maternal age (at birth, years)	30.5 ± 3.5
Gestational weight gain (kg)	11.68 ± 4.37

The data here is mean ± SD.

### SITAR results

**Fig. 1:**
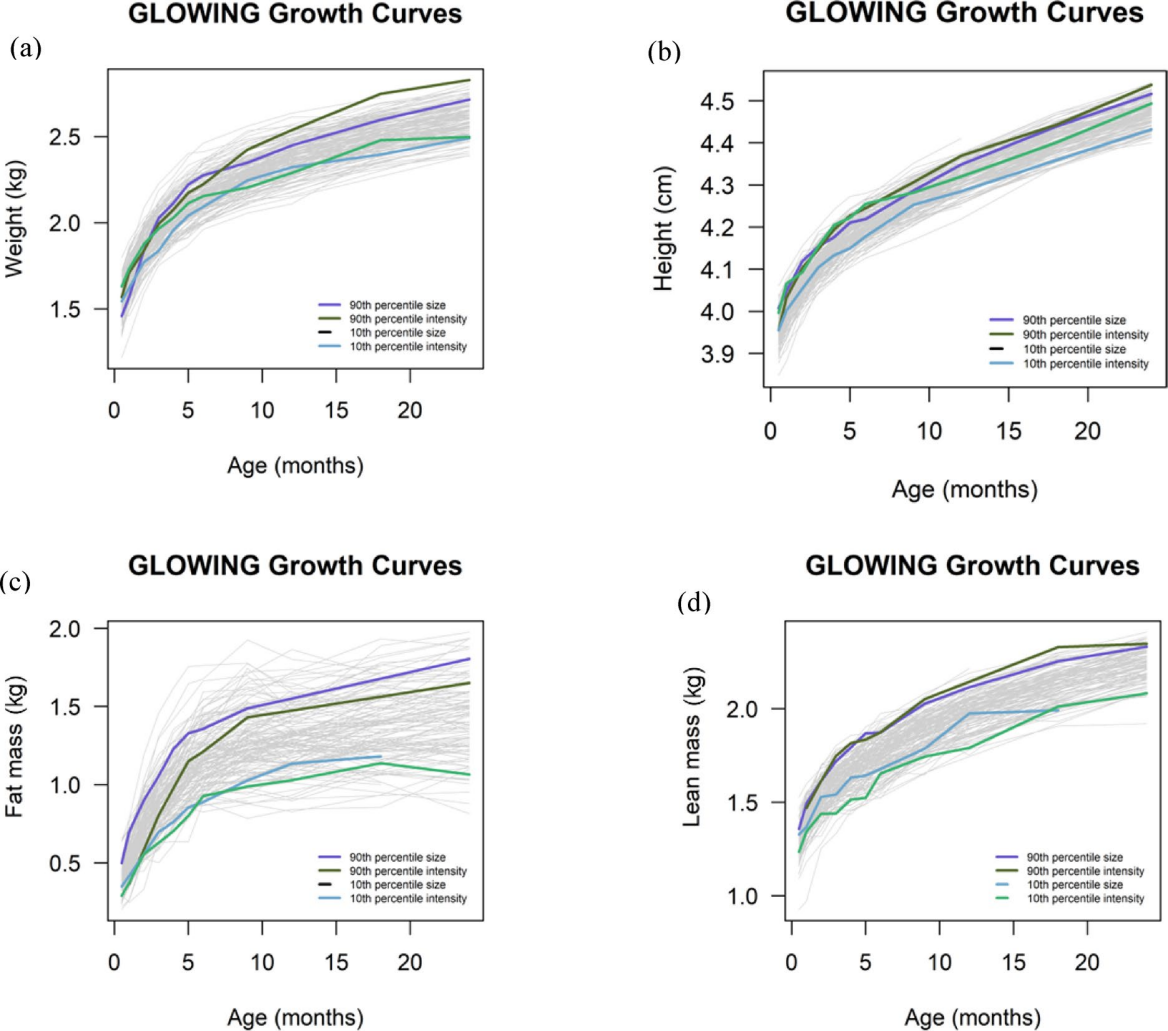
Trajectories of anthropometric measurements for weight (**a**), height (**b**), fat mass (**c**) and lean mass (**d**) across the follow-up period, while highlighting the 90th (purple and dark green) and 10th (light blue and green) percentiles of the size and intensity values for each measure to help visualize differences in the extremes of these parameters in our study sample.

We used SITAR to characterize individual variation in average size and growth velocity (intensity in anthropometric characteristics over the follow-up period (Fig. 1). For weight and height all participants had at least 5 datapoints available across the follow-up period, while for fat mass and lean mass they had a minimum of 4 datapoints. Supplement Table 1 summarizes the median values and missing visits for weight, height, fat mass, and lean mass across different time points.

To understand the association between the intensity and size parameters with placental GAA, we conducted linear regression analysis (Tables 2 and 3), while adjusting for child sex, maternal age at birth, maternal education, gestational weight gain. In a second model, we also adjusted for cell type proportions, specifically trophoblasts, stromal, hoffbauer, endothelial, nRBC, and synctiotrophoblasts. As a secondary analysis, we additionally adjusted our models for maternal BMI at enrollment. Inclusion of maternal BMI as a covariate did not alter the associations observed between placental gestational age acceleration and postnatal growth outcomes, suggesting that our findings are robust to differences in maternal size.

**Table 2:** Association between the velocity (Intensity) of growth measures in children over the first 2 years of life and the gestational age acceleration obtained from placental tissues.

Parameter	Model 1			Model 2		
	Estimates	CI		Estimates	CI	
Weight	− 0.014*	− 0.028	− 0.0001	− 0.012	− 0.027	0.002
Height	− 0.006	− 0.016	0.004	− 0.004	− 0.015	0.006
Fat mass	− 0.041*	− 0.072	− 0.010	− 0.042*	− 0.074	− 0.010
Lean mass	− 0.007	− 0.019	0.005	− 0.006	− 0.018	0.007

Model 1: Parameters adjusted for child sex, maternal age at birth, maternal education, maternal, gestational weight gain.

Model 2: Parameters adjusted for model 1 + cell type proportions of Trophoblasts, Stromal, Hoffbauer, Endothelial, nRBC, Synctiotrophoblast.

*Indicates significant p value at < 0.05.

**Table 3:** Association between the magnitude (Size) of growth measures in children over the first 2 years of life and the gestational age acceleration obtained from placental tissues.

Parameter	Model 1			Model 2		
	Estimates	CI		Estimates	CI	
Weight	− 0.007	− 0.028	0.014	− 0.009	− 0.031	0.012
Height	0.000	− 0.007	0.007	− 0.001	− 0.008	− 0.005
Fat mass	− 0.057*	− 0.100	− 0.013	− 0.063*	− 0.108	− 0.017
Lean mass	0.007	− 0.011	0.025	0.007	− 0.012	0.025

Model 1: Parameters adjusted for child sex, maternal age at birth, maternal education, gestational weight gain.

Model 2: Parameters adjusted for model 1 + cell type proportions of: Trophoblasts, Stromal, Hoffbauer, Endothelial, nRBC, Synctiotrophoblast.

*Indicates significant p value at < 0.05.

In our primary models, we observed a statistically significant association of GAA with intensity for weight (95% CI [− 0.03, − 0.001]), indicating that increased placental age acceleration relates to slower weight gain in the first two years. Although the direction of effect remained negative when cell proportions were included in the model, the effect was somewhat weaker and confidence intervals crossed the null. Interestingly, GAA was also associated with the intensity of fat mass gain in both our primary model (95% CI [− 0.08, − 0.02]) and after controlling for cell composition (95% CI [− 0.08, − 0.02]) indicating that increased placental age acceleration is related to a slower rate of fat mass gain through the first two years of life. We did not observe an effect of GAA on height intensity or on lean mass intensity in either model. Similar to the observed associations with the intensity parameter, GAA was also associated with average fat mass in both our primary model (95% CI [− 0.11, − 0.03]) as well as after controlling for cell composition (95% CI [− 0.11, − 0.03]). Thus, we observed that increased GAA is related to lower average fat mass over time. There was no effect of GAA on the size parameters for weight, height, and lean mass.

## Discussion

We analyzed the longitudinal measurements of children’s growth trajectories over the first two years of life using two parameters of SITAR, size and intensity. The intensity parameter characterizes the rate at which an individual’s growth trajectory progresses along the age scale. While the size parameter represents variations in mean body measurements among individuals over time. This report shows that higher placental GAA was associated with slower weight gain and fat mass gain over time, as well as reduced average fat mass over the follow-up period. However, no significant effect of GAA was observed on height and lean mass through 24 months of age.

To date, studies of GAA have predominantly used cord blood samples, with DNA methylation patterns in cord blood shown to accurately predict GAA^13^. However, our study highlights the use of placental tissues for analyzing GAA, which may offer an alternate reflection of the intrauterine environment and a critical organ for moderating fetal growth^25^. The placenta plays a pivotal role in fetal development, influencing nutrient transport, immune modulation, and hormonal regulation^26,27^. DNA methylation within the placenta is crucial for the development and the regulation of trophoblasts, which are fundamental determinants of fetal growth outcomes^28^. Therefore, studying placental GAA may provide deeper insights into placental perturbations or in-utero environmental disruptions that impact childhood growth trajectories.

To our knowledge, this is the first study to examine the association between early childhood growth patterns and GAA measured from placental tissue. Previous research in cord blood has shown that higher GAA was associated with larger birthweight and birth length of the child^29^. Their focus on cord blood GAA and birth outcomes, versus our focus on the placenta and postnatal growth trajectories, may account for differences in the observed results. Another study investigated the association between GAA with height and weight trajectories from birth to 10 years using cord blood samples from the Avon Longitudinal Study of Parents and Children (ALSPAC)^30^. Their findings indicate that infants with a one-week increase in GAA were born heavier and taller, with these differences diminishing after around 9 months of age. However, from approximately 5 years of age onward, the relationship between GAA and weight reversed, with GAA associated with lower weight, and this association became more pronounced with advancing age. Alternatively, a study by Simpkin, et al. utilized a lifespan epigenetic clock^31^ instead of a gestational epigenetic clock and found that at age acceleration at birth was correlated with higher average fat mass from infancy to adolescence (0–17 years)^32^. The timepoints and tissue types in these other studies differ from ours. Thus, it is not surprising that some of our reported results conflict with theirs. However, it is notable both our study and the ALSPAC study that examined GAA^30^, found that higher GAA was associated with lower average weight after infancy. We provide a very granular focus on early growth patterns with 11 time points from 2 weeks postnatal to 24 months, which is when the most rapid growth occurs and when inflections to a growth trajectory can have substantial consequences on prospective growth potential.

In our study, we observed a negative association between GAA and the rate of weight and fat mass change up to 24 months, as well as reduced average fat mass. While we compare our results to other studies of perinatal age acceleration above, the primary distinction between these studies and ours lies in the use of placental tissue and growth data across a larger number of follow-up visits but over a shorter period of time. Our findings underscore the potential of placental DNA methylation-derived GAA to provide early insights into growth trajectories. While the SITAR method has predominantly been utilized to understand weight and height patterns^22,33^, our study demonstrates its utility in interpreting fat mass and lean mass growth trajectories as well.

Our study explores the complex relationship between placental-GAA and child weight gain, as well as fat mass gain, giving insights into early growth trajectories. The negative association between higher GAA and reduced weight gain, along with a decrease in fat mass and slower fat mass accumulation, provides novel insights into the interplay between prenatal factors and postnatal growth patterns. It is essential to understand these findings within the broader scope of children’s health and development. Weight gain and fat mass accumulation during early childhood serve as important indicators of nutritional adequacy and metabolic health^34^. Therefore, while higher GAA may be related to reduced weight and fat mass gains, these outcomes may also reflect responses to prenatal exposures or perturbations to the in-utero environment. Additionally, reduced fat mass gain can provide protection against excessive weight gain and reduces the risk of childhood obesity^35^. The interrelationships between placental GAA with postnatal growth and adiposity are undoubtedly complex and require further study. We speculate that the observed association between higher placental GAA and reduced weight and fat mass gain in early childhood may reflect a compensatory response to in-utero stressors or early maturation of the placenta. An accelerated placental epigenetic age may indicate adaptive processes such as altered nutrient sensing or endocrine signaling that aim to protect the fetus during gestation but may also program more conservative postnatal growth patterns. However, additional research is needed to formally test these hypotheses.

The study has several limitations that should be considered when interpreting the findings. Firstly, the relatively small sample size of 152 dyads, predominantly health pregnancies, and a narrow window of gestational ages at birth (37–42 weeks) may restrict the generalizability of the results. Additionally, the follow-up duration, up to 24 months postnatally, does not fully capture the long-term effects of placental gestational age acceleration (GAA) on growth and health outcomes throughout childhood. One limitation of our analytic approach is the use of the SITAR model, which, while effective at capturing individual variability in size, tempo, and velocity of growth, relies on smoothed, single-phase growth curves. As such, SITAR may not fully capture more complex, biphasic, or multiphasic growth patterns that can occur during early childhood. Future studies could consider alternative or complementary modeling approaches that allow for greater flexibility in characterizing non-linear or segmented growth trajectories, particularly in populations with high biological variability. In spite of these limitations, our findings highlight the importance of comprehensive monitoring of childhood growth trajectories, not just weight and height but also fat mass and lean mass accumulation, in the context of GAA. Additionally, our results emphasize the potential use of placental DNA methylation-derived GAA as a perinatal factor that may help to identify children at risk of altered growth patterns. Future research examining the specific molecular pathways linking higher age acceleration to reduced weight gain and fat mass accumulation will provide valuable insights of early growth patterns and their effects for long-term health outcomes.

## Conclusion

Overall, our findings suggest that placental gestational age acceleration may have differential effects on body composition, with weight and fat mass gain being reduced, while height, and lean mass appear to be unaffected. Further research with larger sample sizes or alternative methodologies may provide additional insights into the interplay between prenatal health and childhood growth patterns.

## Supplementary Information

Below is the link to the electronic supplementary material.

Supplementary Material 1

## Data Availability

Data that are presented in this publication have been deposited in NCBI’s Gene Expression Omnibus and are accessible through GEO Series accession number GSE288358.

## Abbreviations

GAA: Gestational age acceleration
GA: Gestational age
EGA: Epigenetic gestational age
DNA: Deoxyribonucleic acid
DNAm: DNA methylation
NIEHS: National Institute of Environmental Health Sciences
BMI: Body mass index
STD: Sexually transmitted disease
QMR: Quantitative nuclear magnetic resonance
CPC: Control placental clock
RPC: Robust placenta clock
SITAR: SuperImposition by translation and rotation
nRBC: Nucleated red blood cells

## References

[1] Burton, G. J. & Fowden, A. L. The placenta: a multifaceted, transient organ. *Philos. Trans. R Soc. Lond. B Biol. Sci.* **370** (1663), 20140066 (2015). 25602070 10.1098/rstb.2014.0066 PMC4305167

[2] Napso, T., Yong, H. E. J., Lopez-Tello, J. & Sferruzzi-Perri, A. N. The role of placental hormones in mediating maternal adaptations to support pregnancy and lactation. *Front. Physiol.* **9**, 1091 (2018). 30174608 10.3389/fphys.2018.01091 PMC6108594

[3] Sferruzzi-Perri, A. N. & Camm, E. J. The programming power of the placenta. *Front. Physiol.* **7**, 33 (2016). 27014074 10.3389/fphys.2016.00033 PMC4789467

[4] Chou, F. S. et al. Exposure to placental insufficiency alters postnatal growth trajectory in extremely low birth weight infants. *J. Dev. Orig Health Dis.* **11** (4), 384–391 (2020). 31581967 10.1017/S2040174419000564

[5] Lewis, R. M. et al. The placental exposome: placental determinants of fetal adiposity and postnatal body composition. *Ann. Nutr. Metab.* **63** (3), 208–215 (2013). 24107818 10.1159/000355222

[6] Arisaka, O., Ichikawa, G., Koyama, S. & Sairenchi, T. Childhood obesity: rapid weight gain in early childhood and subsequent cardiometabolic risk. *Clin. Pediatr. Endocrinol.* **29** (4), 135–142 (2020). 33088012 10.1297/cpe.29.135 PMC7534524

[7] Belsky, D. W. et al. Polygenic risk, rapid childhood growth, and the development of obesity: evidence from a 4-decade longitudinal study. *Arch. Pediatr. Adolesc. Med.* **166** (6), 515–521 (2012). 22665028 10.1001/archpediatrics.2012.131 PMC3534740

[8] Nummela, S. R. et al. Weight gain in infancy and markers of cardiometabolic health in young adulthood. *Acta Paediatr.* **111** (8), 1603–1611 (2022). 35366015 10.1111/apa.16349 PMC9543448

[9] Woo, J. G. Infant growth and Long-term cardiometabolic health: a review of recent findings. *Curr. Nutr. Rep.* **8** (1), 29–41 (2019). 30729427 10.1007/s13668-019-0259-0

[10] Jain, V. & Singhal, A. Catch up growth in low birth weight infants: striking a healthy balance. *Rev. Endocr. Metab. Disord*. **13** (2), 141–147 (2012). 22415299 10.1007/s11154-012-9216-6

[11] Singhal, A. Long-Term adverse effects of early growth acceleration or Catch-Up growth. *Ann. Nutr. Metab.* **70** (3), 236–240 (2017). 28301849 10.1159/000464302

[12] Ong, K. K., Ahmed, M. L., Emmett, P. M., Preece, M. A. & Dunger, D. B. Association between postnatal catch-up growth and obesity in childhood: prospective cohort study. *BMJ* **320** (7240), 967–971 (2000). 10753147 10.1136/bmj.320.7240.967 PMC27335

[13] Knight, A. K. et al. An epigenetic clock for gestational age at birth based on blood methylation data. *Genome Biol.* **17** (1), 206 (2016). 27717399 10.1186/s13059-016-1068-z PMC5054584

[14] Bohlin, J. et al. Prediction of gestational age based on genome-wide differentially methylated regions. *Genome Biol.* **17** (1), 207 (2016). 27717397 10.1186/s13059-016-1063-4 PMC5054559

[15] Lee, Y. et al. Placental epigenetic clocks: estimating gestational age using placental DNA methylation levels. *Aging (Albany NY)*. **11** (12), 4238–4253 (2019). 31235674 10.18632/aging.102049 PMC6628997

[16] Mayne, B. T. et al. Accelerated placental aging in early onset preeclampsia pregnancies identified by DNA methylation. *Epigenomics* **9** (3), 279–289 (2017). 27894195 10.2217/epi-2016-0103 PMC6040051

[17] Polinski, K. J. et al. Epigenetic gestational age and the relationship with developmental milestones in early childhood. *Hum. Mol. Genet.* **32** (9), 1565–1574 (2023). 36617164 10.1093/hmg/ddac302 PMC10117157

[18] Daredia, S. et al. Prenatal and birth associations of epigenetic gestational age acceleration in the center for the health assessment of mothers and children of Salinas (CHAMACOS) cohort. *Epigenetics* **17** (13), 2006–2021 (2022). 35912433 10.1080/15592294.2022.2102846 PMC9665122

[19] Fortin, J. P., Triche, T. J. Jr. & Hansen, K. D. Preprocessing, normalization and integration of the illumina humanmethylationepic array with Minfi. *Bioinformatics* **33** (4), 558–560 (2017). 28035024 10.1093/bioinformatics/btw691 PMC5408810

[20] Teschendorff, A. E. et al. A beta-mixture quantile normalization method for correcting probe design bias in illumina infinium 450 k DNA methylation data. *Bioinformatics* **29** (2), 189–196 (2013). 23175756 10.1093/bioinformatics/bts680 PMC3546795

[21] Andres, A., Gomez-Acevedo, H. & Badger, T. M. Quantitative nuclear magnetic resonance to measure fat mass in infants and children. *Obes. (Silver Spring)*. **19** (10), 2089–2095 (2011). 10.1038/oby.2011.215 21779094

[22] Cole, T. J., Donaldson, M. D. & Ben-Shlomo, Y. SITAR–a useful instrument for growth curve analysis. *Int. J. Epidemiol.* **39** (6), 1558–1566 (2010). 20647267 10.1093/ije/dyq115 PMC2992626

[23] Riddell, C. A., Platt, R. W., Bodnar, L. M. & Hutcheon, J. A. Classifying gestational weight gain trajectories using the SITAR growth model. *Paediatr. Perinat. Epidemiol.* **31** (2), 116–125 (2017). 28075023 10.1111/ppe.12336 PMC5323272

[24] Ohuma, E. O., Bassani, D. G., Qamar, H., Yang, S. & Roth, D. E. A novel development indicator based on population-average height trajectories of children aged 0–5 years modelled using 145 surveys in 64 countries, 2000–2018. *BMJ Glob. Health* **6** (3) (2021). 10.1136/bmjgh-2020-004107 PMC7925247 33648981

[25] Beaumont, R. N. et al. Genome-wide association study of placental weight identifies distinct and shared genetic influences between placental and fetal growth. *Nat. Genet.* **55** (11), 1807–1819 (2023). 37798380 10.1038/s41588-023-01520-w PMC10632150

[26] Jansson, T. & Powell, T. L. Role of placental nutrient sensing in developmental programming. *Clin. Obstet. Gynecol.* **56** (3), 591–601 (2013). 23703224 10.1097/GRF.0b013e3182993a2e PMC3732521

[27] Murphy, V. E., Smith, R., Giles, W. B. & Clifton, V. L. Endocrine regulation of human fetal growth: the role of the mother, placenta, and fetus. *Endocr. Rev.* **27** (2), 141–169 (2006). 16434511 10.1210/er.2005-0011

[28] Koukoura, O., Sifakis, S. & Spandidos, D. A. DNA methylation in the human placenta and fetal growth (review). *Mol. Med. Rep.* **5** (4), 883–889 (2012). 22294146 10.3892/mmr.2012.763 PMC3493070

[29] Khouja, J. N. et al. Epigenetic gestational age acceleration: a prospective cohort study investigating associations with familial, sociodemographic and birth characteristics. *Clin. Epigenetics*. **10**, 86 (2018). 29983833 10.1186/s13148-018-0520-1 PMC6020346

[30] Bright, H. D. et al. Epigenetic gestational age and trajectories of weight and height during childhood: a prospective cohort study. *Clin. Epigenetics*. **11** (1), 194 (2019). 31842976 10.1186/s13148-019-0761-7 PMC6916215

[31] Horvath, S. DNA methylation age of human tissues and cell types. *Genome Biol.* **14** (10), R115 (2013). 24138928 10.1186/gb-2013-14-10-r115 PMC4015143

[32] Simpkin, A. J. et al. The epigenetic clock and physical development during childhood and adolescence: longitudinal analysis from a UK birth cohort. *Int. J. Epidemiol.* **46** (2), 549–558 (2017). 28089957 10.1093/ije/dyw307 PMC5722033

[33] Cole, T. J. & Mori, H. Fifty years of child height and weight in Japan and South korea: contrasting secular trend patterns analyzed by SITAR. *Am. J. Hum. Biol.* **30** (1) (2018). 10.1002/ajhb.23054 PMC5811819 28833849

[34] Vieira, S. A. et al. Influence of weight gain rate on early life nutritional status and body composition of children. *ScientificWorldJournal* **2014**, 616108 (2014). 25538953 10.1155/2014/616108 PMC4236901

[35] Kim, J. & Lim, H. Nutritional management in childhood obesity. *J. Obes. Metab. Syndr.* **28** (4), 225–235 (2019). 31909365 10.7570/jomes.2019.28.4.225 PMC6939706

