# Supplementary material for: Association between placental epigenetic age acceleration and early postnatal growth patterns
Source: Sci Rep. 2025 Aug 12;15:29597. doi: 10.1038/s41598-025-13951-y (PMC12343783; doi:10.1038/s41598-025-13951-y)
Supplement: Supplementary file 1 — Supplementary Material 1 [file 41598_2025_13951_MOESM1_ESM.docx]

Supplement Table 1. Median Values and Missing Visits for Weight, Height, Fat Mass, and Lean Mass Across Different Time Points

|  | **Weight (kg)** | | **Height (cm)** | | **Fat Mass (kg)** | | **Lean Mass (kg)** | |
| --- | --- | --- | --- | --- | --- | --- | --- | --- |
| **Visit (month)** | **Median** | **Missed Visits** | **Median** | **Missed Visits** | **Median** | **Missed Visits** | **Median** | **Missed Visits** |
| 0.5 | 3.70 | 2 | 51.58 | 2 | 0.46 | 9 | 2.68 | 9 |
| 1 | 4.36 | 2 | 53.75 | 2 | 0.65 | 7 | 3.05 | 7 |
| 2 | 5.38 | 1 | 57.3 | 1 | 1.06 | 4 | 3.61 | 4 |
| 3 | 6.05 | 3 | 60.00 | 3 | 1.40 | 13 | 3.97 | 13 |
| 4 | 6.68 | 7 | 62.50 | 8 | 1.67 | 15 | 4.30 | 15 |
| 5 | 7.27 | 3 | 64.30 | 3 | 1.94 | 11 | 4.55 | 11 |
| 6 | 7.72 | 4 | 65.90 | 4 | 2.17 | 7 | 4.78 | 7 |
| 9 | 8.88 | 3 | 70.10 | 3 | 2.53 | 13 | 5.54 | 13 |
| 12 | 9.86 | 7 | 74.10 | 7 | 2.61 | 27 | 6.33 | 27 |
| 18 | 11.30 | 13 | 81.00 | 14 | 3.00 | 35 | 7.52 | 35 |
| 24 | 12.52 | 19 | 86.75 | 19 | 3.22 | 44 | 8.41 | 44 |
